# Extending the Limits of Wireless Power Transfer to Miniaturized Implantable Electronic Devices

**DOI:** 10.3390/mi8120359

**Published:** 2017-12-12

**Authors:** Hugo Dinis, Ivo Colmiais, Paulo Mateus Mendes

**Affiliations:** CMEMS, University of Minho, 4800-058 Guimarães, Portugal; icolmiais@gmail.com (I.C.); paulo.mendes@dei.uminho.pt (P.M.M.)

**Keywords:** wireless power transfer, inductive coupling, midfield, far-field, ultrasound, biological energy harvesting

## Abstract

Implantable electronic devices have been evolving at an astonishing pace, due to the development of fabrication techniques and consequent miniaturization, and a higher efficiency of sensors, actuators, processors and packaging. Implantable devices, with sensing, communication, actuation, and wireless power are of high demand, as they pave the way for new applications and therapies. Long-term and reliable powering of such devices has been a challenge since they were first introduced. This paper presents a review of representative state of the art implantable electronic devices, with wireless power capabilities, ranging from inductive coupling to ultrasounds. The different power transmission mechanisms are compared, to show that, without new methodologies, the power that can be safely transmitted to an implant is reaching its limit. Consequently, a new approach, capable of multiplying the available power inside a brain phantom for the same specific absorption rate (SAR) value, is proposed. In this paper, a setup was implemented to quadruple the power available in the implant, without breaking the SAR limits. A brain phantom was used for concept verification, with both simulation and measurement data.

## 1. Introduction

The landscape of the medical electronics field is rapidly changing, due to the continued development of new and miniaturized sensors, actuators, processors and packaging technologies. Performance, reliability, and functionalities, such as data collection and wireless communications of electronic medical implants are on the rise. However, these perks are inevitably accompanied by an energy requirement that must be met for proper device operation.

Long-term and reliable powering within the human body has been a major challenge since the first implantable pacemaker was developed in the 1960s [1]. Batteries have been used as the first option for long operation time, but their high volume, limited lifetime, and miniaturization limits have been an issue for further miniaturizing medical implants that aim to reach locations with severely limited available space. Even when batteries fit the solution, battery replacement surgeries are usually unavoidable, which can be harmful to the patients and load health services’ waiting lists. This energy drama may be solved combining three vectors: making implants more energy efficient (low-power electronics), improving the power storage mechanisms, or using alternative energy capture solutions. The promising powering methodologies for miniaturized implants are energy harvesting from the environment, such as thermal gradients or muscle movements, and wireless power transfer, using dedicated links based on different power transfer mechanisms.

This paper presents a review of representative state of the art miniaturized implantable medical devices with wireless power capabilities, and aims to evaluate and compare powering methods that are used in the most advanced devices of the academic field. After such analysis, a new proposal is made, to multiply the power that can be transferred wirelessly, without going above health safety levels.

## 2. Implantable Medical Devices and the Need for Miniaturized Wireless Power Transfer (WPT) Approaches

Implantable medical devices have been undergoing a constant and rapid miniaturization, which is a consequence of recent developments in integration and fabrication technologies. Powering ultra-small devices has become a major concern, as batteries take up unavailable volume and impose limited device lifetimes. Even though some applications’ requirements can be met with passive devices, such as the wireless pressure monitor presented in [2], most applications require active devices that need a power source to operate. Alternative powering methods are being studied by several researchers and are evolving at a quick pace, but these haven’t yet met the desired criteria for some of the smallest implants. An example was reported in Oxley et al. [3]. The authors presented an endovascular neural interface for neural recording which does not require craniotomy for implantation, avoiding brain trauma and inflammatory responses. This is achieved by guiding the implant through veins until the brain, as cerebral veins lie in sulcal folds. It consists of a stent-electrode array, which takes advantage of the existent knowledge on stent technology to deliver the integrated electrodes into the desired location for chronical measurements.

This prototype is a novel approach to deep brain electrical signal recording, yet data transmission relies on a wire. Wire fatigue was verified in chronic implantation, which hinders the durability of the implant. The authors have proposed wireless power and data transmission as a solution to this issue, but stated that wireless technology at the time of developing the prototype was still too large for endovascular approaches.

If chronical and long-lasting implantation is not a requirement—i.e., for short time monitoring of a particular set of biological parameters—transient electronics can be used. The pressure sensor presented in [4] is a prime example of such biodegradable devices. It consists of a silicon nanomembrane strain gauge, poly(lactic-co-glycolic acid) (PLGA), silicon dioxide and nanoporous silicon, which are all biodegradable materials that are absorbed within 30 h of accelerated exposure to aqueous solutions.

As these devices are designed to be implanted for a limited amount of time and aim for biodegradability, a battery to power them is not a suitable solution. The transient nature of these devices allows biodegradable molybdenium wires to connect the sensing module of the implant—which is commonly placed in hard-to-reach and volume-restrictive locations—to a processing and communication module in a larger area. This eliminates the need for surgical retrieval procedures and allows data and power links. Nevertheless, there is a need for a head stage to be in contact with the wires, to read data and supply power to the device. An alternative approach could be the use of said biodegradable wires as antennas for power and data links, allowing for increased freedom of use by the test animal, reduced risk of infection and easier access to deeper locations in an animal’s body.

Bioresorbable wiring presents itself as a viable communication path for a small implant. Nevertheless, it is only suitable for devices with limited and well defined lifetimes. Furthermore, head stages and other forms of external communication modules and power sources are preferably avoided in clinical use, due to imposed constraints to the user and the risk of infection. For chronical implantation, miniaturized wireless methods appear to be the only viable and universally accepted powering option.

## 3. Wireless Power Transfer (WPT) in Recent State of the Art Devices

Ground-breaking wireless powering methods have been shown to be in high demand and an essential part of modern miniaturized electronic implants. Even though alternative approaches have been discussed, they are only applied in a minority of cases. The focus of this review is on state of the art implantable devices. The smallest, bordering technological limits, health safe, innovative and representative devices with wireless power capabilities will be reviewed. A trend line in most used wireless power transfer (WPT) technologies will be established, and their pros and cons discussed.

### 3.1. Inductive Coupling

One of the most common methods of power transmission to medical devices is inductive coupling (IC), as it suffers the least losses by absorption in body tissue at lower frequencies. This method has been previously used to power cochlear implants, total artificial hearts and neural implants, among others. Inductive coupling is based on electromagnetic induction on conductors, where an alternating current in a transmitting coil generates a magnetic field, which couples with a receiving coil and generates an electromotive force that can be used to power the implanted device [5]. Despite its popularity, this method has drawbacks, such as coil decoupling due to misalignment, since it requires rigorous positioning of transmitter and receiver coils [6,7,8]. Moreover, the range of inductive coupling complies with exponential decay, a near-field phenomenon, meaning that the external coil must be close to the implant.

**Figure 1:**
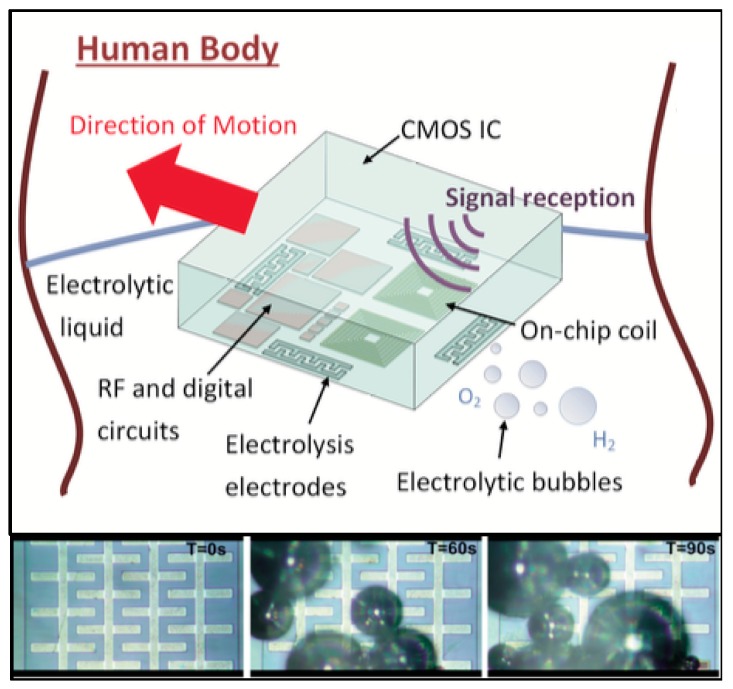
Representation of the remote-controlled device and its movement mechanism (**top**); with photographs of electrolysis bubble formation over time (**bottom**). Reproduced with permission from [9].

In their 2014 publication [9], Hsieh et al., developed a remote-controlled device with a propulsion mechanism based on gas pressure from electrolytic bubbles that can move at a rate of 0.3 mm/s. Electrolysis electrodes, placed in its four directions, and an electrical current cause electrolysis of the surrounding water, creating micro-bubbles of oxygen and hydrogen which propel the device and are later absorbed by the human body. It was possible to define the electrodes in which the electrolysis would occur and, consequently, steer the device. A previously reported device [10] of this type requires a strong permanent magnetic field to generate the Lorentz forces responsible for movement. Hsieh’s device removed this constraint and took this technology one step closer to real world applications. A representation of the device and a time-lapse of electrolytic bubble formation are presented in Figure 1.

This technology is potentially suitable for drug delivery, tumor scanning and bio-testing, among other applications, especially in hollow organs, such as the bladder and the stomach, and even in blood. Power and commands are delivered to the device via a 10 MHz inductive coupling link with a 15.2% measured efficiency for a 0.5 cm coil separation. On-chip coils are present and make up a considerable portion of its area. Total power consumptions are 207.4 μW and 180 μW for electrolysis voltages on 2 V and 1.3 V, respectively.

In [11], a subcutaneously implantable wireless optogenetic device was presented. It is two orders of magnitude smaller than the previous state of the art device. Optogenetics consists of the use of light to manipulate neural function, thus modulating the behavior of the subjects. Previous systems resorted to optic fibers to deliver light to neurons, yet these tethered approaches impose several constraints on experimentation, as the optic fibers must be correctly placed and handled and the animal subject must be placed in a limited environment, thus limiting the experiments that can be performed. The authors report that, recently, there have been attempts to deliver light wirelessly, eliminating the tethers. Nevertheless, the devices’ volumes and requirements of headstages containing electronic components limit their application in most scenarios, as deep brain regions cannot be reached and animal behavior can be influenced by the headstage.

The optogenetic implant developed by the authors consists of a power receiving coil, an LED and a rectifying circuit, and its size and weight ranges from 10 to 25 mm^3^ and 20–50 mg, depending on the target of operation. As reported, such a small device can be implanted in the animal prior to experiments and allows free movement. In order to power such a small device without interfering with the animals, the authors developed a cage which acts as a resonance cavity. It couples 1.5 GHz electromagnetic energy to the tissue of a mouse through its surface. Unlike traditional IC links, energy is located in a rat. The dielectric properties and dimension of the animal cause the electromagnetic field generated by the surface of the cage to resonate with its body, thus concentrating energy in the rat and making it available all over its body.

For a time averaged input power of 3.2 W (20% duty cicle) at the surface of the cage, 5.6–15.7 mW were delivered to the implant. Despite the great results reported, this approach presents considerable drawbacks. Variable animal masses and volumes require a tuning of the cage in order for resonation to occur with that animal. Furthermore, despite eliminating the need for an external power source on the body and an external coil to be precisely aligned with the implant’s coil, the need for a specially designed and tuned ground surface for implant powering poses a severe drawback for real life application of this technology in other scenarios.

Inductive coupling has been the backbone of WPT mechanisms for a good number of years, yet its limitations are causing newer and less restrictive technologies to take over. Even though high power levels can be delivered, the need for either a carefully aligned pair of coils or a dedicated, individual-tuned power source, such as in previously reported cases, is a limitation that must be overcome. The next sections of this paper will present other WPT technologies used in state of the art devices, aiming to provide readers with knowledge of what is out there and what the current and future options are, concerning WPT.

### 3.2. Midfield

The heterogeneous and conductive nature of biological tissue, combined with the exponential decay of the near-field, render near-field WPT methods, such as inductive coupling, unsuitable for powering a deeply implanted miniaturized device.

According to [12], power transfer in the midfield (MF) region can surpass the aforementioned challenges, and this occurs around a wavelength away from the source. The midfield region allows power flow lines to be manipulated with an interference, focusing them in a specific spot [13].

**Figure 2:**
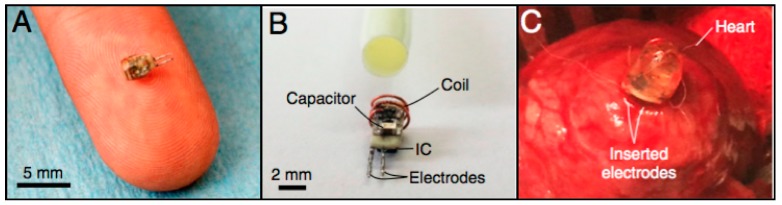
(**A**) Miniaturized wireless pacemaker on a human finger for scale; (**B**) device composition, displaying its coil, capacitor and inductive coupling (IC); and (**C**) an implanted device. Reproduced with permission from [12].

In [12], a catheter-implantable wireless cardiac pacing device (Figure 2) was presented. It has a cylindrical shape, with a diameter of 2 mm, height of 3.55 mm and weight of 70 mg. Like the power measurement probe, it consists of a power receiving coil, RF-DC converter, electrodes and an IC for pulse control.

**Figure 3:**
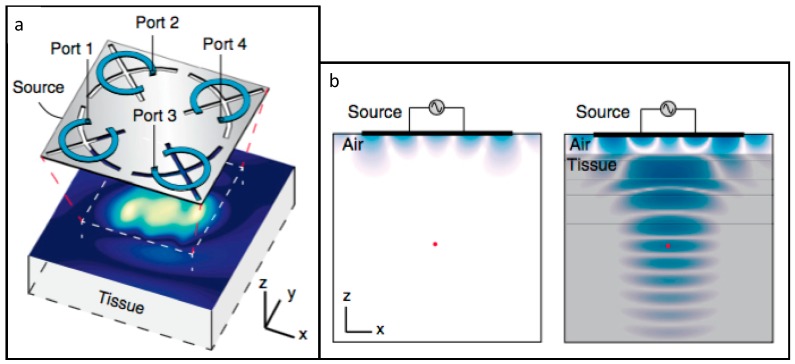
(**a**) Schematic of the 1.6 GHz midfield power source placed above tissue and the magnetic field on the skin surface; and (**b**) the resulting magnetic field in air and couple into tissue. Reproduced with permission from [12].

In order to power the device in the midfield region, the authors developed a patterned metal plate, presented in Figure 3a. As represented in Figure 3b, the presence of biological tissue below the patterned metal plate allows for energy to be coupled to the tissue, thus reducing losses by radiating into air. The cardiac pacing device was inserted into a rabbit heart for cardiac pacing. It was achieved with a 1 W portable power source, located 4.5 cm away from the implant and resorting to the patterned metal plate.

To measure the power level that can be transmitted to a deeply implanted device, a custom probe was developed by the authors. It consisted of a power receiving coil, RF-DC converter, a LED and an IC capable of translating power level to pulse frequency. This device was implanted in the brain, 5 cm away from the metal plate, and in the heart, 5.5 cm away from the same structure. When coupling the maximum amount of energy permitted by specific absorption rate (SAR) regulations, 2.2 mW was recovered in the first scenario, and 1.7 mW in the second one. For a 500 mW input power, SAR levels were an order of magnitude below the safety threshold and 195 μW and 200 μW were transferred in each scenario, respectively, which corresponds to an efficiency of approximately −34 dB, or 0.04%.

Agrawal et al. [13] proposed an improvement over the previous WPT system. Its core is a phased surface, comprised of a flexible set of rings that are loaded by capacitors, responsible for coupling the energy with the tissue and focusing the power at a specific point. The phased surface’s flexibility allows it to conform to the shape of the body, thus improving energy coupling.

Similarly to the previous paper, a miniaturized device with a coil, an RF-DC converter and an LED were designed, to experimentally validate the power transfer efficiency. After encapsulation, it weighed 20 mg and had a 12 mm^3^ volume.

With an 800 mW output from the phased surface, 0.45 mW was delivered to the device when it was implanted 4.2 cm deep into multilayered porcine tissue, corresponding to a 5.6%, or −32.5 dB power transfer efficiency, a substantial increase over the previous paper’s results. The absence of phase control capacitors in the phased surface translated in a five-fold power reduction, which highlighted the importance of phase control. The phased surface is also capable of coupling over 96% of its output power into tissue, reducing power loss through radiation in air.

A 1.5 mm diameter and 5 mm long pacemaker was designed to test the suitability of the phased surface in real applications. Power inputs at the phased surface, ranging from 34 mW to 216 mW, were enough to successfully pace the heart at different locations (right atrium and left ventricle, respectively), which are well below the established safety thresholds.

Two cardiac pacemakers that are much smaller than commercially available solutions, both powered in the MF, were presented. This powering method was proven to be enough for deeply implanted microdevices to operate when requested. Nevertheless, this technique requires an external component to be aligned with the implant for proper operation, as well as a portable and wearable power source, much like inductive coupling solutions.

### 3.3. Far-Field

Optogenetics has revolutionized the study and control of neural functions, yet no method of truly untethered and unconstrained application has been presented. The device from [11] has been the closest to a truly untethered approach, yet a subject-specific lattice cage was required, as previously demonstrated.

**Figure 4:**
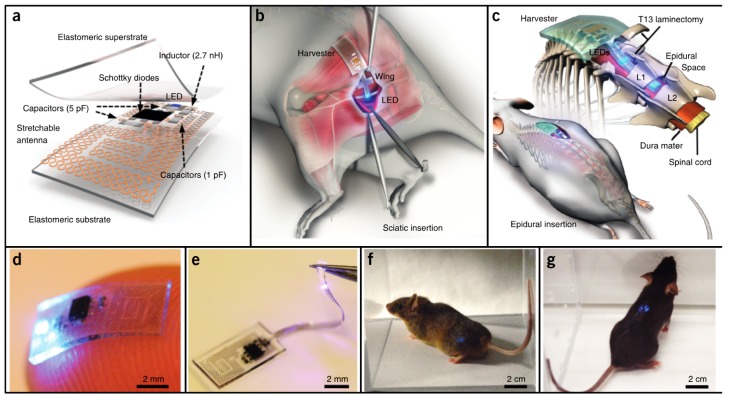
(**a**) Schematic of the optoelectronic system and (**b**,**c**) implantation site. (**d**,**e**) Photographs of the functioning device; and (**f**,**g**) mouse implantation with the LED operating. Reproduced with permission from [14].

Park et al. [14] developed soft and biocompatible wireless optoelectronic systems, capable of molding to their environment and sustaining natural motions, allowing operation in previously inaccessible places. The proposed device, presented in Figure 4a,d,e, consists of an RF antenna, an RF-DC rectifier and an LED for neural modulation. A representation of implantation of the device is given in Figure 4b,c, and photographs of mice implanted with the device in Figure 4f,g.

The antenna, which is only 3 × 3 mm^2^, can receive power at 2.34 GHz. The design is relatively unaffected by physiological antenna strains, as an experimental exaggerated strain resulted in a 12% efficiency decrease. The LED could produce 100 μW of optical power by being activated with 2 W transmitters, located up to 20 cm away from the implant, which removes the constrains of midfield and inductive coupling powering methods.

**Figure 5:**
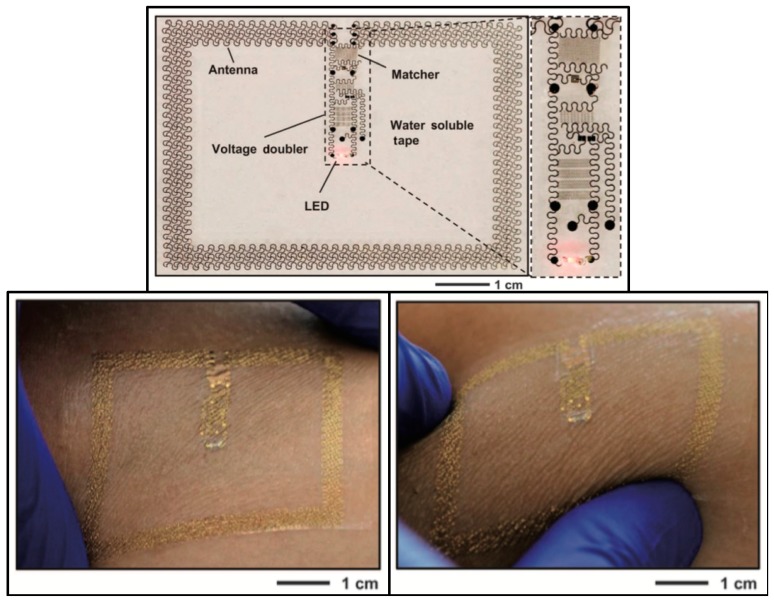
Photograph of the wearable wireless device with the LED turned on (**top**) and photographs of the device attached to skin and squeezed (**bottom**). Reproduced with permission from [15].

A device with similar characteristics and design was proposed in [15]. Despite targeting wearable application instead of implantable ones, it served to further illustrate that the aforementioned design has application potential in diverse areas and for different goals. The 5.4 × 4 cm^2^ device is presented in Figure 5. It can power an LED with 32 mW from an external 15 W transmitter, connected to an 11 dBi antenna, radiating at 1 GHz, 1.5 m away from the target.

Far-field (FF) WPT allows for truly untethered power link designs, with transmitters being placed at a considerable distance from the implant and the patient. Even though overall link efficiency is lost with free-space propagation losses, power at the receiver can be kept the same, as long as the transmitter output power can be increased without surpassing SAR limits.

### 3.4. Ultrasound

It has been stated in the bibliography that conventional WPT techniques, such as inductive coupling and far-field RF powering, are inefficient at powering small implants located deep into the body, mostly due to the small size of the receiving antenna and the energy dissipation that occurs in the conductive biological tissue.

Ultrasounds (US) have been proposed as a power delivery method. Low wave velocity and consequent improved coupling and efficiency, combined with the ability to focus energy in specific spots, and low propagation loss [16], makes this an appealing approach. Furthermore, the food and drug administration (FDA) defined maximum allowed power via US (7.2 mW/mm^2^) is around two orders of magnitude higher than that of RF waves (10–100 μW/mm^2^) [17].

In [17], Charthad et al., presented a 7.8 × 4 mm^2^ implantable proof-of-concept device with a 1 × 1 mm^2^ piezoelectric transducer for power acquisition, an IC for AC-DC rectification, data recovery and transmission, a capacitor for energy storage, and a loop antenna for a ultra-wideband (UWB) bi-directional data link. The piezoelectric transducer recovered 400 μW from an incident acoustic density of 1 mW through 3 cm of chicken meat, showing an efficiency of 5.6%. The device has potential to be used in stimulation or monitoring applications.

**Figure 6:**
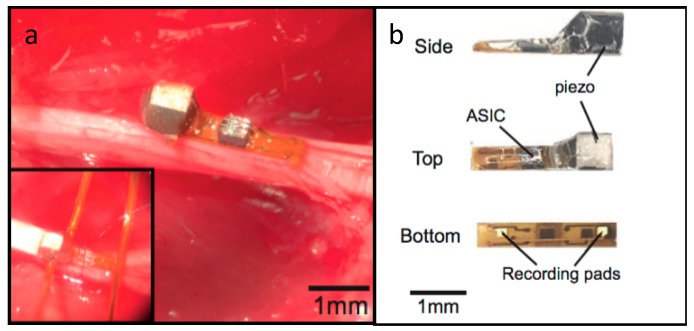
(**a**) Nervous system recorder placed in the sciatic nerve of a rat and (**b**) layout of the device. Reproduced with permission from [16].

A peripheral nervous system single-neuron recorder is presented in [16]. It consists of a piezocrystal (0.75 × 0.75 × 0.75 mm^3^) for powering and communication purposes, a transistor and two recording electrodes. The whole device measures around 0.8 × 3 × 1 mm^3^ and is presented in Figure 6.

The working principle of the device is centered around its transistor. External US pulses are either converted into electrical currents by the implant’s piezoelectric crystal, which is supplied to the transistor, or reflected to the external transmitter. The two recording electrodes modulate the transistor’s gate, according to the potential across the electrodes, which results in a change in the amount of current flowing through the crystal. This translates into a modulation of the reflected signal, which encodes the neuron voltage captured by the electrodes for later reconstruction. The authors validated the device and acquired both ENG and EMG signals from the peripheral nervous system of rats.

Ultrasound WPT has been proposed to overcome some limitations of RF-based power transmission. By having smaller wavelengths and slower propagation speeds in biological tissue, energy loss is reduced and better coupling with a small receiver is possible, which results in a higher system efficiency. Nevertheless, US transmitters need to be placed in direct contact with skin to couple the waves with tissue, which results in a reduced freedom for the patient or test subject, as power sources need to be near the device, much like in inductive coupling systems.

### 3.5. Discussion

The previous section of this paper presented state of the art implantable electronic devices, whose power demands are met with WPT mechanisms. These range from inductive coupling to ultrasounds, including midfield and far-field transfer. The previously reported devices’ characteristics are summarized in Table 1. WPT link distance, antenna/transducer size, received power at the implant and link efficiency are presented, along with the calculated power density (obtained by dividing the received power by the antenna/transducer size).

Inductive coupling implants can provide high power levels, with Montgomery’s method [11] being the one with the highest power output and density, and also provide high efficiencies, as shown by Hsieh’s device [9]. Nevertheless, this method is limited by its range and by the fact that it requires precise tuning of the WPT elements (in the case of the first device) and careful alignment between transmitters and receivers (second device).

Midfield regime power transfer devices boast larger link distances, as expected, and power densities, comparable to those of Hsieh’s device. Nevertheless, efficiency is lower than that of IC links. In comparison to the previous kind of devices, these allow for greater implant depths at the cost of link efficiency. Like IC links, careful alignments between equipment are required, as well as an external power source connected to the transmitting element of the system, which can hinder the subject’s mobility and freedom.

Far-field WPT provides, as expected, the largest link ranges at the expense of link efficiency. Furthermore, lower power densities are observed, which are due to SAR regulation limits.

Ultrasound links have proven to be the most efficient of all the studied methods, as shown in Charthad’s paper [17]. The second highest power density was registered in the same publication—all this while operating at one seventh of the FDA power limit. On the negative side, US are not efficient when facing against interfaces of tissues with different impedances, such as different organs and muscle. Furthermore, attenuation in liquids and bone is high, which can limit its application and efficiency. Finally, the transmitting US transducer needs to be placed in direct contact with the skin of the subject, due to the air-skin impedance mismatch, which gives birth to the same mobility and freedom issues as inductive coupling and midfield power transfer.

In conclusion, US appears to be the best WPT solution when carrying external components in contact with the skin is not an issue to the subject or patient, and bearing in mind the tissue path that the US waves must travel, i.e., whether there will be plentiful tissue interfaces, bone or fluids in the path. If this is the case, then perhaps MF WPT can be used alternatively, as both methods allow focusing of the energy hotspot. When mobility and absence of carry-on equipment is a requirement, FF WPT takes the lead, at the expense of link efficiency and power level.

## 4. Extending the Available Power

From the previous section of this paper, it is possible to conclude that power availability at the implant level is low, and more power-hungry devices will have trouble receiving enough energy for their operation. A complementary power source can be used for such devices, and biological energy harvesting (EH) presents itself as a possible solution, allowing for power to be received from the outside and harvested from inside the human body.

### 4.1. Biological Energy Harvesting

Energy harvesting techniques consist of retrieving useful amounts of energy from the device’s environment, and use it for its own operation. These techniques have the potential for acting as unlimited sources of power, as they mostly rely on biologically renewable energy sources, such as muscle movement, vibrations, or glucose.

Energy harvesters for implantable devices can be divided in several categories, according to their power source, such as thermoelectric generators, biomechanical energy, solar power, biofuel and RF energy harvesters [18].

Dagdeviren et al. [19] developed a piezoelectric energy harvester from heart motion. Being a large organ with high displacement in natural motions, it is a good source of biomechanical energy. Nevertheless, a harvester must be designed, so to not induce constraints on the organ’s motion.

The presented device consists of a piezoelectric material, placed between two electrodes (a mechanical energy harvester (PZT MEH) for power generation from heart movement), an AC-DC rectifier and a battery. The authors validated the device by implanting it in a bovine heart.

Experimental results demonstrate that enough energy can be generated to continuously power a cardiac pacemaker, during over 20 million operation cycles. The device’s electrical power output, per unit area of PZT, was measured at a maximum of 0.18 μW/cm^2^.

Zheng et al. [20] reported on a biodegradable triboelectric nanogenerator. This device is composed solely of biodegradable material, and is thus degraded and absorbed in the body, therefore not requiring surgery for retrieval after its lifetime is over.

The device converts biomechanical energy into electrical energy by friction between materials that originate a voltage differential (triboelectric effect). A power density of 32.6 mW/m^2^ was reported by the authors, and the potential for a fully implantable solution was also documented, upon integrating the device with an electrode and a wireless communication module.

The endocochlear region, located in the inner ear, produces and maintains a 70–100 mV voltage potential, called the endocochlear potential (EP), much like a battery. The EP can be used as a renewable power source for small and power efficient electronics, as demonstrated by Mercier et al. [21,22]. A 9 × 11 mm^2^ implantable device was presented by the authors. Although the device was not tested with a full implantation, electrodes were placed in the endocochlear region of a guinea pig, to harvest the EP, and connected to the device. It was able to extract a minimum of 1.12 nW for the 5 h duration of the experiment, which was enough to transmit EP measurements every 40–360 s, at 2.4 GHz.

Biofuel cells have been well documented in the recent past, especially with the works of E. Katz et al. [23]. These systems generate electricity through oxidation reactions inside the physiological medium of biological tissue. Zebda et al. [24] developed a glucose biofuel cell, based on nanotube-matrix bioelectrodes and implanted it in a rat. The 0.24 mL volume device can power an LED and a digital thermometer, with a power output of 161 μW/mL.

Biological EH is a promising renewable power source for implantable devices, is completely tether-free and takes advantage of biologically available energy sources. Nevertheless, very small amounts of power can be harvested; therefore, this powering method’s usefulness is limited to devices that possess extremely low power consumptions, or as a complementary energy supply for a device that receives power from a dedicated source.

### 4.2. Multipath Far-Field WPT

Despite all the differences between the previous devices, whether in application or WPT method, all have a common point. A single power transmission path is used between the source and the implanted device. To maximize the amount of transferred power to the device, a multipath approach is proposed. As discussed before, US and FF appear to be the best WPT solutions. Since multiple tissue interfaces are expected between the air and the implantable device, and completely tetherless operation is desirable. FF was the selected WPT mechanism for the multipath approach, as it is less affected by interfaces than ultrasound waves and does not require external components attached to the skin.

The proposed multipath approach consists of the use of multiple transmitting antennas in the FF regime, making use of multiple paths for energy inside the human body, which can be tuned to focus on the region of the implant. With this, more power can be delivered to the implant with the same SAR value, as power is distributed more evenly throughout the tissue, rather than being focused on a single path.

**Figure 7:**
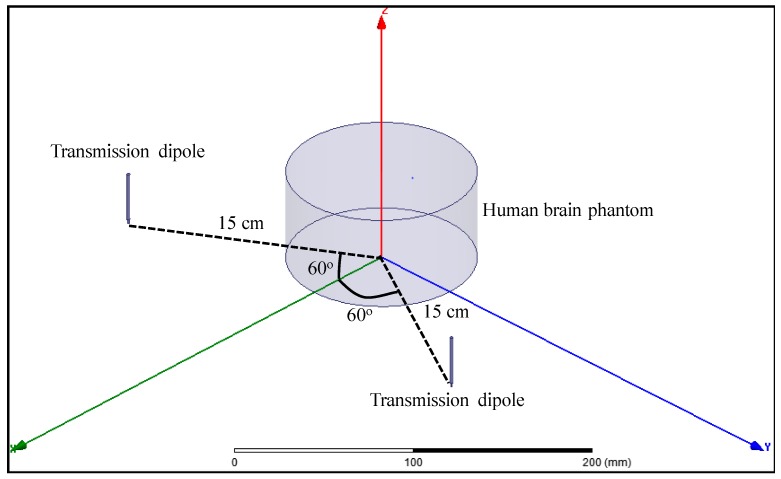
High frequency structure simulator (HFSS) model of the proposed multipath FF WPT approach.

A multipath far-field WPT approach is proposed and validated with Ansys high frequency structure simulator (HFSS) simulations. A model, consisting of a cylinder with the dielectric properties of human brain at 2 GHz was designed, and two 2 GHz half-wavelength dipole antennas were placed one wavelength away from the center of the brain phantom, both at a 60° angle from the symmetry plane, as shown in Figure 7.

**Figure 8:**
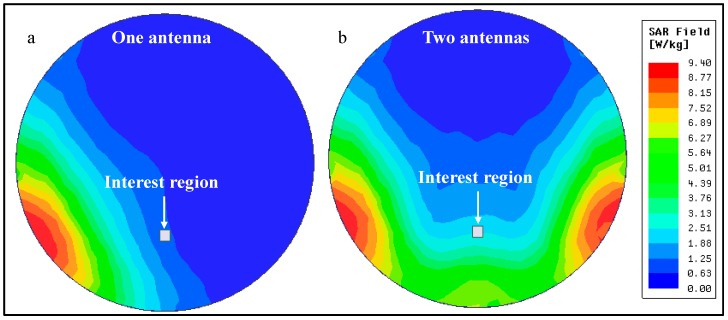
Distribution of 10-g averaged SAR over the human body phantom for: (**a**) one transmission dipole active with 10.2 W of power; and (**b**) both dipoles transmitting 11.2 W each.

This model was simulated with either both dipoles activated, or only one of them. To keep comparisons as fair as possible, the output power of the transmitting antennas was adjusted, so that the SAR value on the human brain phantom was the same in both scenarios. For one active transmission antenna, an output power of 10.2 W was selected for a maximum SAR value of 9.4 W/kg (averaged over 10 g of tissue). With this, a maximum Poynting’s vector magnitude of 10.9 W/m^2^ was registered at the selected interest region, given by the square highlighted in Figure 8. For two transmission antennas, outputs of 11.2 W per antenna resulted in the same maximum SAR value, as confirmed by Figure 8a,b. In this scenario, a maximum Poynting vector magnitude of 41.2 W/m^2^ was registered in the same region of interest, an increase of nearly four-fold in available power in the same region.

**Figure 9:**
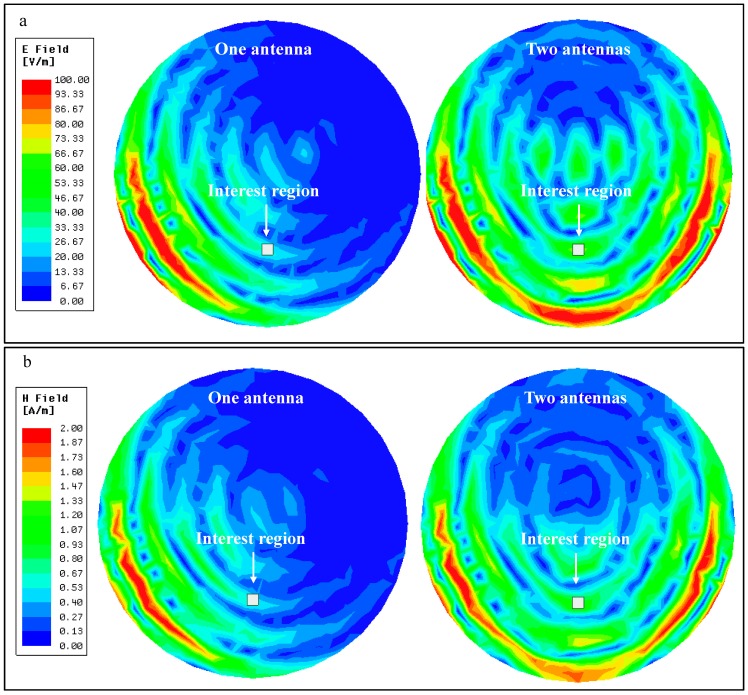
(**a**) Electric field and (**b**) Magnetic field distribution in the human brain phantom for one and two transmitting antennas.

A two-fold increase in received power was easily anticipated, as the total power being transmitted by the antennas has approximately doubled (from one antenna transmitting 10.2 W to two antennas at 11.2 W each). The remaining increase in available power (measured by Poynting’s vector) occurs because the fields transmitted by each antenna meet and combine in amplitude inside the brain phantom, as shown in Figure 9, which translates into twice the power at that same location.

From the previous figure, it is possible to see that, at the interest region, the E-field increases from around 30 V/m when one antenna is used, to around 60 V/m when two antennas are transmitting. The H-field presents a similar behavior, increasing from around 0.6 A/m to 1.2 A/m. When using two transmission antennas, both the E-field and the H-field double in value in the region of interest. As available power is a product of these fields, it quadruples, as previously demonstrated.

**Figure 10:**
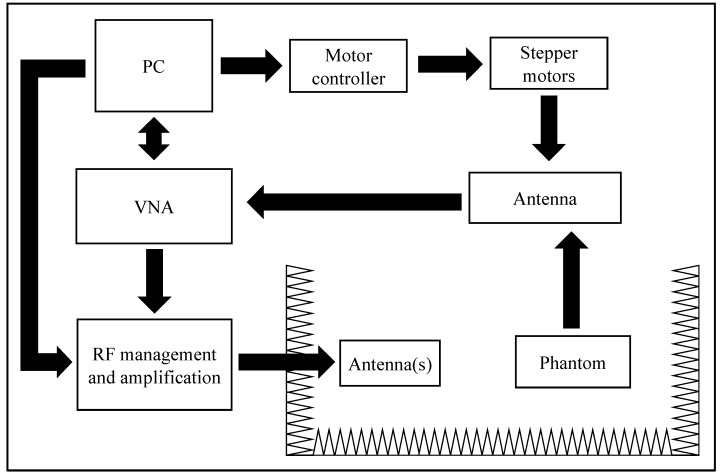
Experimental setup for power measurement inside a liquid phantom.

To validate the simulated results, the experimental setup of Figure 10 was developed. A LabView script running on a PC controlled the placement of a receiving dipole antenna inside the liquid phantom through a two stepper motor, while simultaneously controlling a vector network analyzer (VNA) (Keysight E5071C, Keysight Technologies, Santa Rosa, CA, USA). The RF management and amplification block was responsible for allowing easy commutation from operation with one and two transmitting antennas. The power measurement was performed inside an anechoic chamber to reduce interference from external elements.

**Figure 11:**
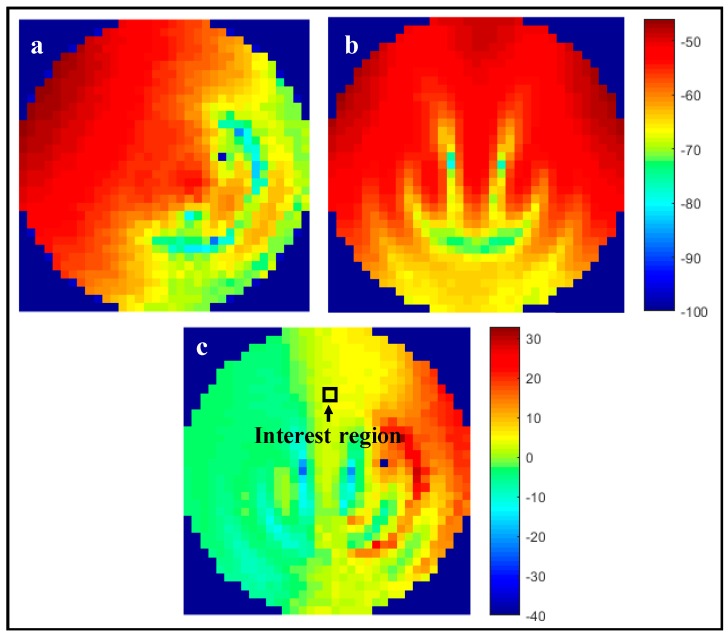
Power distribution inside the human brain phantom for (**a**) one transmitting antenna; (**b**) two transmitting antennas; and (**c**) difference of (**a**,**b**).

For the experimental measurements, the total transmitted power was kept constant, therefore, and according to simulated results, a two-fold increase in available power at the interest region was expected. For the single-path scenario, one of the transmitting dipole antennas was disconnected, and the remaining one radiated 0 dBm. In the multi-path scenario, each of the two dipoles transmitted −3 dBm, leading to the same source total power as the first case. The measurement results are presented in Figure 11.

Analyzing the interest region, which coincides with the one from the simulation, it is possible to determine that there is a ~3.0 dB power increase in the two antenna versus the single antenna scenario, which agrees with simulation results, as available power at the interest region doubles for the same total input power.

## 5. Conclusions

A review of state of the art implantable electronic medical devices with wireless power capabilities was presented. These devices obtain power via inductive coupling, midfield and far-field ultrasounds and biological energy harvesting. From the analysis of the selected publications, ultrasounds can be the most effective power transfer mechanism, but their application is limited, due to the need to have transducers in contact with the skin and to the high attenuation of ultrasound waves in tissue interfaces and bone. Far-field WPT is another solution, as there is no need for external components to be worn or carried by the subject or patient. A far-field multipath WPT approach was presented and validated with simulation and experimental data. Numerically, for the same SAR value, a dual-path WPT system can deliver approximately four times more power than the traditional one antenna far-field WPT system. Experimentally, it was verified that for the same output power, the multipath approach doubled the available power at the interest region, which confirms and validates simulation data.

## Figures and Tables

**Table 1:** Summary of implantable devices’ wireless power transfer (WPT) link characteristics.

Ref.	Type	Depth (cm)	Frequency	Antenna (mm^2^)	Power (mW) @ Implant	Efficiency (%)	Power Density
[9]	IC	0.5	10 MHz	2.3	0.2	15.2	0.09 mW/mm^2^
[11]	IC	<3	1.5 GHz	6	15.7	0.5	2.62 mW/mm^2^
[12]	MF	4.5	1.6 GHz	9.4	0.2	0.04	0.02 mW/mm^2^
[13]	MF	4.2	1.6 GHz	5.3	0.45	0.06	0.08 mW/mm^2^
[14]	FF	20 * 0.5 **	2.34 GHz	9	0.1 ***	-	-
[15]	FF	150 *	1 GHz	2160	32	0.2	0.01 mW/mm^2^
[16]	US	-	1.85 MHz	0.56	-	-	-
[17]	US	3	1 MHz	1	0.36	5.7	0.36 mW/mm^2^

* In air; ** Mouse tissue; *** Optical power at LED.
